# Correlation of Bone Material Model Using Voxel Mesh and Parametric Optimization

**DOI:** 10.3390/ma15155163

**Published:** 2022-07-25

**Authors:** Kamil Pietroń, Łukasz Mazurkiewicz, Kamil Sybilski, Jerzy Małachowski

**Affiliations:** Institute of Mechanics and Computational Engineering, Faculty of Mechanical Engineering, Military University of Technology, gen. Sylwestra Kaliskiego 2, 00-908 Warsaw, Poland; kamil.pietron@wat.edu.pl (K.P.); lukasz.mazurkiewicz@wat.edu.pl (Ł.M.); jerzy.malachowski@wat.edu.pl (J.M.)

**Keywords:** bone, mechanical properties, material model correlation, optimization, FEA, validation

## Abstract

The authors present an algorithm for determining the stiffness of the bone tissue for individual ranges of bone density. The paper begins with the preparation and appropriate mechanical processing of samples from the bovine femur and their imaging using computed tomography and then processing DICOM files in the MIMICS system. During the processing of DICOM files, particular emphasis was placed on defining basic planes along the sides of the samples, which improved the representation of sample geometry in the models. The MIMICS system transformed DICOM images into voxel models from which the whole bone FE model was built in the next step. A single voxel represents the averaged density of the real sample in a very small finite volume. In the numerical model, it is represented by the HEX8 element, which is a cube. All voxels were divided into groups that were assigned average equivalent densities. Then, the previously prepared samples were loaded to failure in a three-point bending test. The force waveforms as a function of the deflection of samples were obtained, based on which the global stiffness of the entire sample was determined. To determine the stiffness of each averaged voxel density value, the authors used advanced optimization analyses, during which numerical analyses were carried out simultaneously, independently mapping six experimental tests. Ultimately, the use of genetic algorithms made it possible to select a set of stiffness parameters for which the error of mapping the global stiffness for all samples was the smallest. The discrepancies obtained were less than 5%, which the authors considered satisfactory by the authors for such a heterogeneous medium and for samples collected from different parts of the bone. Finally, the determined data were validated for the sample that was not involved in the correlation of material parameters. The stiffness was 7% lower than in the experimental test.

## 1. Introduction

The aim of the undertaken work was to determine the basic stiffness parameters for various ranges of bone tissue density using the results of experimental studies and optimization based on the genetic algorithm. The proposed methodology of the procedure was tested using a single bovine bone, which was selected due to its structure being similar to human bones, high availability, and large dimensions, which facilitated the preparation of a larger number of samples from just one bone.

**Figure 1:**
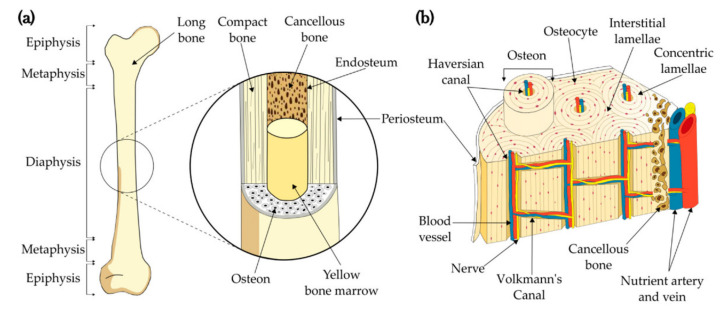
Bone structure: (**a**) long bone and (**b**) microstructure [1].

Most bones in living organisms are supporting structures, with the exception, among others, of teeth and auditory ossicles. Bones are made of bone tissue that is formed during development and growth. They adapt to the transferred loads, so their structures are quite varied. Bone has a hierarchical structure and, from the point of view of the mechanics of a solid, at the macroscopic level, it is a composite material made of two types of bone tissue: compact, termed cortically and spongy-trabecular [1]. In general, it is surrounded by the periosteum, which contains osteoblasts that perform a regenerative and protective function. Compact tissue is responsible for carrying loads and transporting nutrients. Inside it, there is a spongy tissue with a porous structure filled with bone marrow. The microstructure of bones is illustrated in detail in Figure 1. It can be seen that bones do not have a simple, layered structure and that each vascular canal is ‘encircled’ by osteons. Only the periosteum of the bone has a typically layered structure.

The bone tests described in the literature can be divided into two main subcategories: one related to determining basic material parameters and the other to determine complete, complex material characteristics. In the first group of studies, the following are determined mainly: Young modulus, Poisson ratio, and tensile, compressive, and bending strength [2,3]. The second group analyses the complex stress states in bones. These works focus, among others, on research on crack development or the determination of anisotropic parameters [4,5,6].

The problem that arises during experimental tests on biological samples is the wide variation in bone strength parameters for different individuals. It is influenced by factors related to the existence of the individual from whom the material was collected, among others, diet, way of burdening the body, past injuries and diseases, age, or gender. In addition, certain errors or lack of care at the stage of sample preparation can also affect bone stiffness. In the literature, several works on the modelling of animal and human bones have been published [2,3,4,5,6,7,8,9,10,11,12,13]. Table 1 presents a comparison of the basic mechanical properties of human bones available in the literature. The comparison shows a very large dispersion of the Young modulus, which confirms the high differentiation of numbers for different individuals.

From a technical point of view, the bone tests described in the literature mainly include classic strength tests, such as uniaxial stretching, uniaxial compression, and three-point and four-point bending [14], supported in the field of measuring deformations by optical methods (video extensometers, laser extensometers, and the digital image correlation system—DIC) [15]. The second group consists of hardness measurements, and methods determining full characteristics using ultrasound, nanoindentation, computed tomography, or magnetic resonance [16]. Many studies available in the literature show a correlation between bone stiffness and bone density. Therefore, using the most common methods for imaging structures at high resolution (computer tomography, CT), techniques for determining the mechanical properties of the structures and materials were developed on the basis of the image analysis based on the Hounsfield scale [9,10] or the grayscale (cone beam computed tomography, CBCT). Both scales basically determine the degree of absorption of the beam by a given object. In both scales, a pixel is assigned a value proportional to the attenuation of X-ray radiation. For the Hounsfield scale, it is defined by the following formula [17]:

(1) $$HU=1000\cdot \;\frac{\mu -\mu_{H_{2}O}}{\mu_{H_{2}O}}$$

where *µ* is the weakening factor for a given substance, $\mu_{H_{2}O}$ is the water weakening factor, and *HU* is an *HU* scale number [3].

However, the approach of modeling bone as a structure with homogenous properties results in a significant averaging of the properties. A high level of modeling accuracy can be achieved by linking the mechanical properties with the local bone density.

Injuries and diseases of the human skeletal system are also a special issue from the medical point of view, e.g., osteoporosis, which is characterized by weight loss and a weakening of the bone structure and is now considered a disease of civilization. In the case of advanced tissue degeneration, existing solutions in medicine enable the implantation of artificial structures that support or perform tissue functions in the human body [18]. An example of such a procedure is the widely used hip arthroplasty [19]. Due to the existing individual differences in the structure of the human body, personalized medicine plays an increasingly important role, allowing the design of personalized implants. Therefore, we need to know the structure, properties, and material parameters of bone tissue. In addition to experimental studies of bones, numerical modeling also plays an important role [20], which is developed in parallel, allowing analysis of the structure and properties of bone tissue while reducing experimental studies [21]. Commonly used numerical models of bone assume its homogeneity and isotropy of the mechanical parameters of the tissue, which is often an oversimplification of the bone structure. Bearing in mind the above fact related to the hierarchical structure of bones, some scientists are developing methods of multi-scale modeling in which modeling allows the bone microstructure to be taken into account and allows the determination of the distributions of the analyzed local strain fields and related strains at the micro- and macroscopic levels.

Currently, the direct voxel model and the discrete smooth model are used to develop numerical models of bone tissues. The voxel model is the most widespread and most widely used [22,23]. On the basis of two-dimensional images obtained from a CT, a volume model is developed in which the smallest unit of volume (voxel) is converted into a hexagonal finite element. The smooth model consists of segmentation and filtering of two-dimensional images and conversion to a CAD model in the .stl format of a three-dimensional model based on computer microtomography, followed by grid application and volume tetrahedrization [15,24].

Using the Hounsfield scale function from radiological imaging, the modulus of elasticity (Young modulus) can be estimated based on the following relation [25]:

(2) $$E=a\cdot \;\rho_{app}{}^{d}\;\;where\;\rho_{app}=c\cdot HU+b$$

(3) $$E=a\cdot {\left(\;c\cdot HU+b\right)}^{d}$$

where $\rho_{app}$ is the apparent bone density (kg/m^3^), *HU* is the Hounsfield greyscale unit, *E* is the Young modulus of bone (MPa), and *a*, *b*, *c*, and *d* are the coefficients determined by empirical research.

In Equation (3), there are four unknowns that should be determined for a given value of the Hounsfield scale in order to determine the Young’s modulus. In principle, these parameters are constant for the entire bone, so by having a larger number of samples with different densities at your disposal, it is possible to determine the values of these parameters with a high level of accuracy.

Numerical optimization is one of the methods for determining the parameters mentioned above. A typical optimization procedure involves the computation of numerous structural variants to evaluate their key responses [26]. Optimization procedures are often based on simplified models of a given problem for a faster computation of multiple variants in a reasonable time and the acquisition of an optimal solution for real-world problems. In recent years, many publications have been published on computational optimization within this field [27,28]. The parameters of the final structure are optimized by changing the parameters of the components or manufacturing parameters [14], and an inverse method is also used, in which the parameters of the constituent materials are derived from the resulting structure properties. This method is mainly used to identify the material properties of non-homogeneous materials, such as composites [29,30], layered structures [26,31], bones [16], or soil [32]. On the other hand, in [33,34] the authors used optimization to identify the parameters of the homogeneous metallic alloy. In most cases, evolutionary algorithms were used to efficiently derive material parameters using a metamodel-based strategy [29,30] or direct optimization without surrogate models [35].

**Figure 2:**
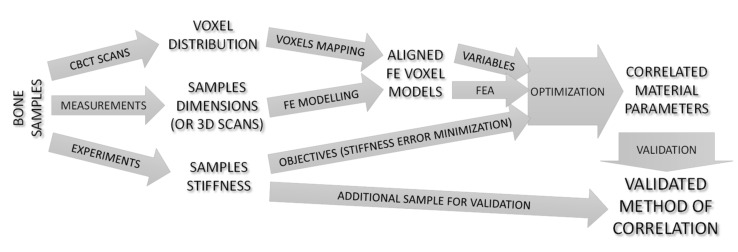
Workflow of the present study.

The authors of this publication, based on the literature stage, decided to verify the possibility of determining the strength parameters for an elastic range of a compact bone based on a simple experimental test and numerical analyses (Figure 2). A novelty in the article is the presentation of a complete algorithm for determining the basic material parameters of bone based on CT scans, simple strength tests, and optimization. This algorithm can be applied to any type of bone, regardless of its structure. In addition, the article presents a detailed methodology for conducting optimizations involving the simultaneous running of multiple numerical tests reflecting different experimental tests to find common parameters describing stiffness. From the point of view of numerical model preparation, the main idea of our method is to map the voxel distribution from tomography, where the mesh is not aligned with the sample, to the new redeveloped model based on physical measurement (or 3D scans, CAD models, etc., if applicable). It is a method that avoids irregularities on the outer walls of the model, improving the convergence of nonlinear analyses and the contact algorithm.

## 2. Materials and Methods

### 2.1. Analyzed Object

**Figure 3:**
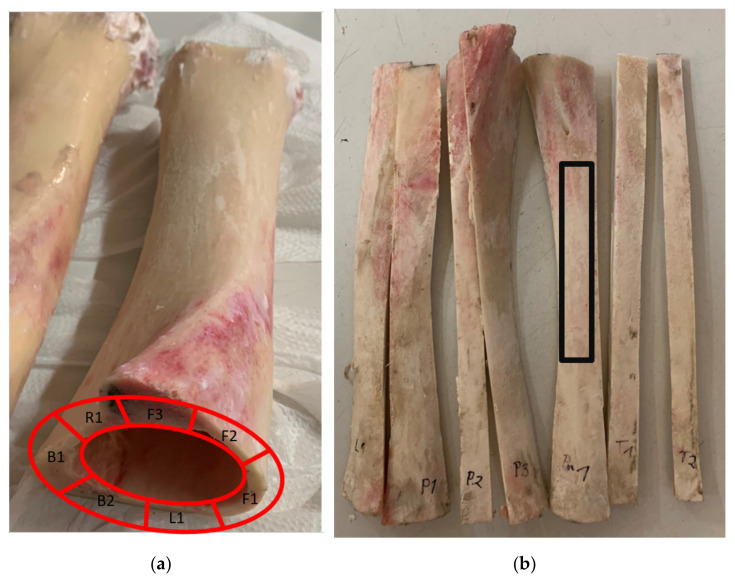
(**a**) Shows the bone after pretreatment with the relevant description, and (**b**) shows the cut pieces of bone that were prepared for cutting out the samples. F1, F2, F3—first, second and third sample from front part of the bone, L1—sample from left side, R1—sample from right side, B1, B2—first and second sample from back part, black box—approximate cutout location of the final sample.

Samples with a rectangular cross-section were tested, which were cut from one bovine thigh bone. The bone was subjected to a preliminary mechanical treatment that involved cleaning the bone from the soft tissue. The next step was to cut the bone, using a band saw, into smaller pieces from which cuboidal samples were cut out later. The bone, cleared from tissues and cut into smaller pieces, is shown in Figure 3. The description of the samples includes the following markings: F—a sample taken from the front part, R—a sample taken from the right side, L—a sample taken from the left side, B—a sample taken from the back part. The number after the letter indicates the sample number.

In the next step, from each ‘strip’ of the central part, a rectangular sample (Figure 3b) was prepared so that the dimensions of the samples corresponded to the adopted dimensions (length 80.0 mm, protruding 12.0 mm, and thickness 8.0 mm). After the entire procedure, the samples were sanded with P120 grit sandpaper to even their outer surfaces. The characteristic dimensions of the samples are presented in Table 2. To identify bone samples in space, one of the corners of each sample was also ground. Samples of both the periosteum and the spongy tissue were ground so that only the central part of the compact tissue was used for testing. Therefore, it did not have the typical periosteal layering.

Finally, the samples were marked and sealed in zip bags. Until the experimental tests were performed, the samples were stored at a temperature of approximately −4 °C.

### 2.2. Experimental Testing

The aim of the performed experimental studies was to determine the load curves as a function of the traverse displacement during the three-point bending of bone samples. A total of six samples (F1, F2, F3, R1, L1, and B1) were tested (Table 2).

**Figure 4:**
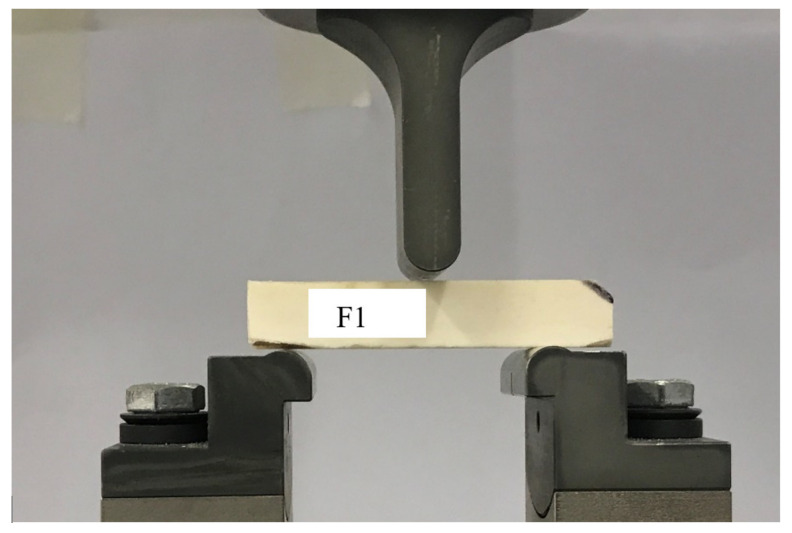
Sample prepared to perform three-point bending.

The way of setting the samples is shown in Figure 4. The samples were placed on supports using a specially designed and 3D-printed positioner so that each sample was always in the same position. The bending test equipment was a set of three-point bending, shown in Figure 4. The lower supports were mounted on a steel beam and had an adjustable spacing of up to 200.0 mm. The supports were made of half-rounds with a radius of R = 5.0 mm. The supports were installed directly on the machine’s traverse in appropriate holders. During each test, the traverse moved to the destruction of the sample at a constant speed of 2 mm/min.

The tests were carried out on a Zwick Roell Kappa 50DS testing machine. The parameters of the testing machine were as follows: maximum load force ±50.0 kN and maximum displacement value 500.0 mm. The testing machine parameters are presented in Table 3. The machine was equipped with manually clamped mechanical jaws and electro-mechanical displacement control.

**Figure 5:**
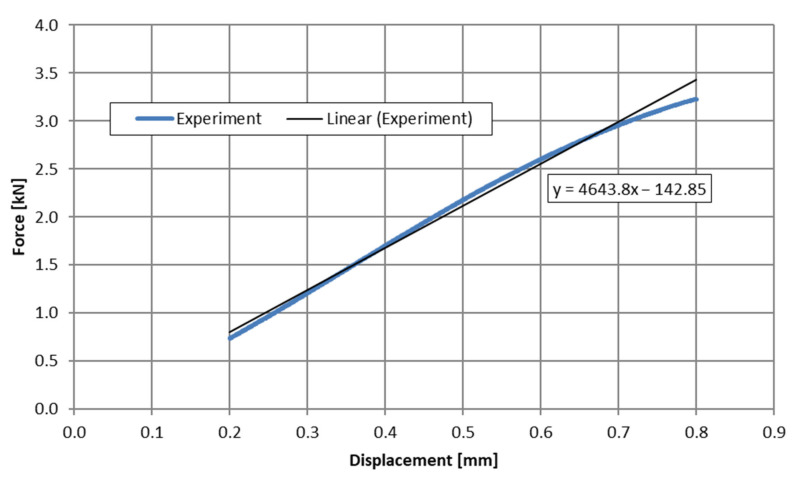
The principle of determining the stiffness (Young’s modulus), $k^{exp}$.

The stiffness of the individual samples was determined during the processing of the results as the slope of the trend line for the linear range of the characteristic (Figure 5) in the range of sample deflection from 0.2 to 0.8 mm.

Based on the results obtained from the experiment, it was observed that the stiffness of individual bone samples ranged from $k_{F2}^{exp}$ = 4080.1 N/mm^2^ for the F2 sample to $k_{L1}^{exp}$ = 5263.7 N/mm^2^ for the L1 sample. The discrepancy in the results was 11.84%.

### 2.3. FE Models Development

#### 2.3.1. CBCT Imaging

**Figure 6:**
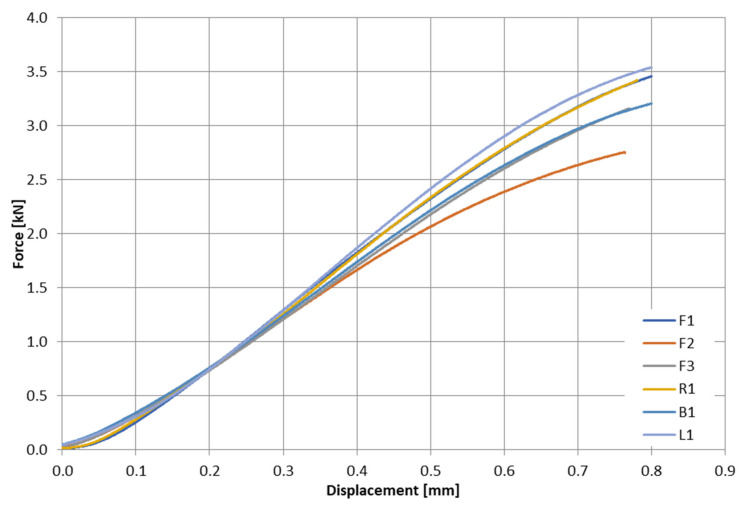
Results of three-point bending test.

Before the strength tests, the bones were scanned on a computed tomography scanner (CBCT) in order to obtain their accurate 3D model, which was broken down by the density of the individual bone phases. Five samples (L1, F1, F2, F3 and B1) were tested and then used in the process of numerical optimization of mechanical properties based on the bending test (Figure 6).

**Figure 7:**
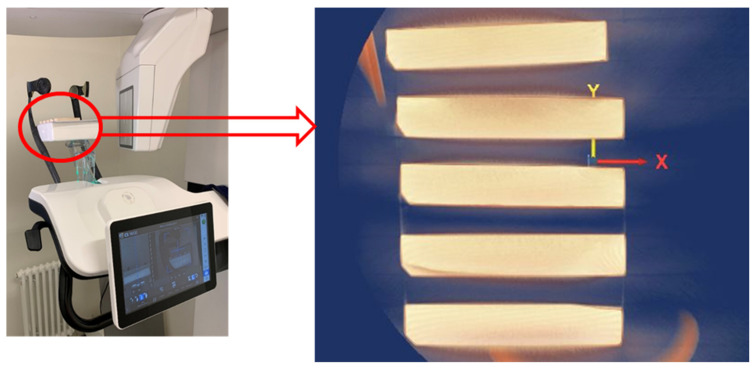
Samples prior to CT scan.

The samples were tested on a Carestream CS9600 CT scanner with an X-ray generator power of 60–120 kV and a tube focal spot equal to 0.3 mm (Table 4, Figure 7). After the examination, a 3D image of the scanned elements was obtained and saved in the DICOM format.

#### 2.3.2. Generation of Voxel Mesh

DICOM images of the scanned bone samples from CBCT were imported into Mimics software developed by Materialize NV. Detailed information on DICOM image parameters is presented in Table 5.

**Table 4:** CT scanner parameters.

Manufacturer	Type	Tube Voltage	Tube Current	Frequency	Tube Focal Spot (IEC 60336)	Total Filtration	Voxel Size
Cerastream Dental LLC, Atlanta, GA, USA	CS9600	60.0–90.0 kV 60.0–120.0 kV (optional)	2.0–15.0 mA	140 kHz	0.3 mm	>2.5 mm eq. Al	75.0 µm minimum

**Figure 8:**
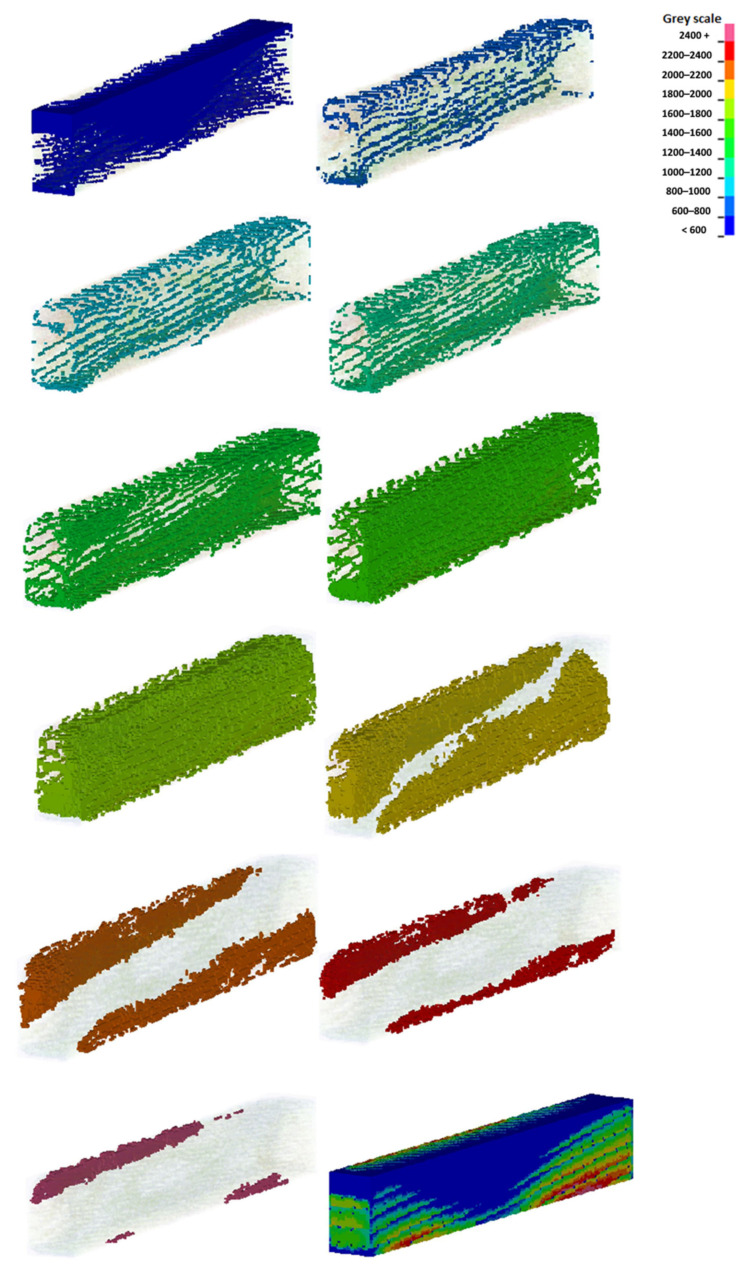
An exemplary division of the voxels sample into groups characterized by a different grayscale range for the K2F3 sample.

**Figure 9:**
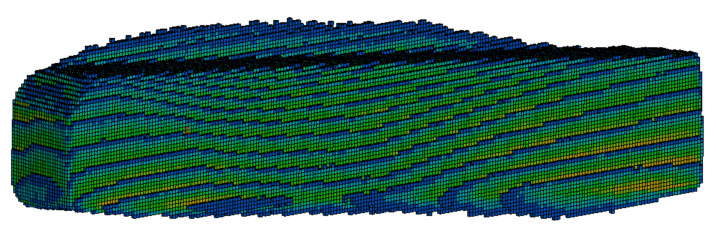
Unequal components of the K2F3 sample immediately after the CBCT scan.

The first step in processing DICOM files was to define the number of grayscale ranges from which voxels would be generated. A greater number of ranges contributes to better accuracy in determining the characteristics of bone stiffness as a function of its density. However, it significantly increases the amount of computing power needed to generate voxels. Ultimately, a decision was made to define 11 ranges. For the selected ranges, masks were created covering the areas characterized by the density in a given range. From the masks, the Mimics system generated voxels (Figure 8), which were then replaced with cubic elements with a side length of 0.3 mm (Figure 9). The coordinate system of the sample was retained from the computer tomograph. All voxel sub-models created with the Mimics software were imported into a single database of the LS-Prepost system and numbered accordingly.

**Figure 10:**
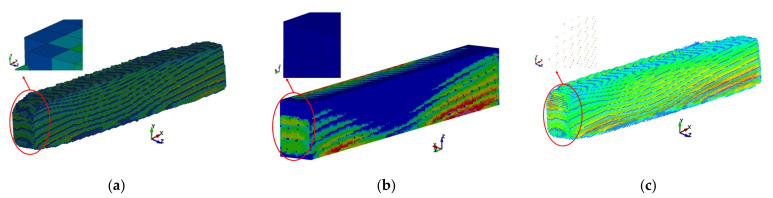
(**a**) Exemplary model of sample no. K2P3, made from tomography consisting of 187,664 voxels, (**b**) midpoints created from tomography, and (**c**) the model of the sample after remeshing 205,128 voxels.

After modeling the samples from the eight-node cubic elements, the walls of the models were very uneven (Figure 9), which significantly hindered the definition of boundary conditions, loads, and further numerical analyses. This was due to the lack of coverage of the axes defined by the walls of the samples by the device’s coordinate system. As a result, the Mimics system, creating voxels, generated them along lines that did not coincide with the walls of cuboidal samples. Therefore, it was necessary to generate new finite elements (remeshing) in the next stage (Figure 10). From the original voxel model, temporary nodes were generated in the center of the volume of each of the 8-node cubic elements independently for each range. Using the coordinates of these nodes, new finite elements were generated for successive ranges, appropriately rotated to the new coordinate system, with axes coinciding with the edges of the samples. The remaining elements, which were outside the scale ranges from 600 to 2400 grayscale units, were placed in the component for the range <600 (Figure 8). For the mesh density of the obtained model, the number of discrete elements in the range from 197,296 to 223,300 was generated. The dimensions of the redeveloped models were based on the physical measurements presented in Table 2.

The determined values show that most elements of the bone on the left side (L1) are in the range of 1600–2200 HU and (F1) 1200–2000, i.e., in the middle range. The largest number of elements of the back of the bone (B1) are in the 1600–2200 range, and the largest number of elements of the right side (R1) are in the 1400–2000 HU range (Table 6). The results presented show that the largest clusters of voxels in the samples are in the range of 1200–2200 HU, that is, in the middle range.

### 2.4. FE Analysis

Computations were performed using the LSTC LS-DYNA^®^ solver (version v11) [36] with the massively parallel processing (MPP) feature, which has been effectively adopted to simulate various problems from different research areas [37,38,39,40]. The simulated problems were characterized through the use of all types of nonlinearity recognized in FEA, including large deformations (geometric nonlinearities) and nonlinear material properties (physical nonlinearity). A computational scheme with a nonlinear implicit (iterative and incremental) method was adopted.

**Figure 11:**
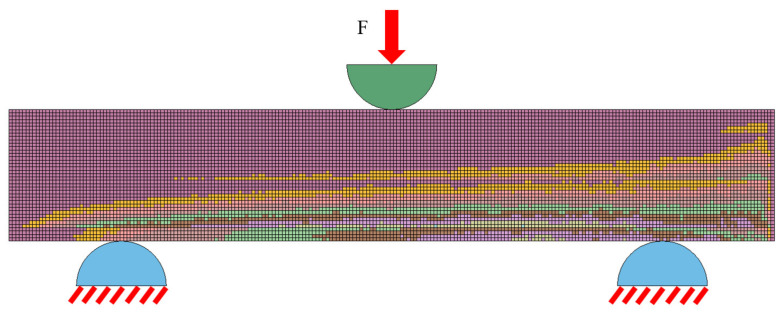
Scheme loading of the FE model of the bone sample characterized by a different system of densities in the three-point bending test.

To replicate the actual tests as closely as possible, the supports and the spindle were modeled using solid elements (HEX8) (Figure 11), and the material parameters were assigned corresponding to the elastic range of steel (E = 210,000.0 MPa, ν = 0.3). The supports were deprived of all degrees of freedom, while for the spindle motion with a constant velocity was defined (in accordance with the experimental tests). A contact was defined between the supports and the sample based on the penalty function method, with the stiffness calculated automatically based on the stiffness of the cooperating components.

### 2.5. Parametric Optimization

The next step was to use the prepared finite models (FE models) to optimize parameters *a*, *b*, *c*, and *d* of Equation (3) and to correlate the results of the experimental studies with the results of the numerical analyses. The optimization is based on simultaneous numerical analyses for many independent numerical models with the same input parameters.

The proposed procedure focused on minimizing the sum of the mean square error of the average stiffness obtained from the FEA for each sample. Due to the linear behavior obtained from the FEA, the average bending stiffness from the numerical analysis was calculated as follows:

(4) $$k^{FEA}=\frac{F_{0.8mm}-F_{0.2mm}}{\Delta d}$$

where $F_{0.8mm}$ and $F_{0.8mm}$ are the forces for 0.8 mm and 0.2 mm deflection, and *∆d* is the deflection increment for stiffness estimation. The stiffness error for a sample was calculated as the difference between the values calculated from the numerical analysis and the experimental test. The error norm is the sum of squared errors for each sample:

(5) $$err=\overset{5}{\underset{i=1}{\sum }}{\left(k_{i}^{FEA}-k_{i}^{Exp}\right)}^{2}$$

where $k_{i}^{FEA}$ and $k_{i}^{Exp}$ represent the stiffness of *i*-sample from the FEA and the experimental test, respectively.

The adopted constraint was the limit of the Young modulus, $E\leq E_{limit}$, based on the literature data [12,13,22,23]. The optimization problem can be described in the following form (see Equation (2)):

(6) $$\min err\left(a,b,c,d\right)\;\mathrm{subjected}\;\mathrm{to}\;E\leq E_{limit}$$

where $err\left(a,b,c,d\right)$ are parameters of the objective function (see Equation (2)) of *a*, *b*, *c*, *d* variables in the following ranges, where *a = <*0.2;0.8*>*, *b = <*1200.0;5000.0*>*, *c = <*2.0;8.0*>*, and *d = <*0.1;2.0*>*.

The ranges of variables corresponding to the domain of the input parameters were selected on the basis of preliminary analyses in such a way that the optimal solution would not be on the border of the search area. Furthermore, the maximum value of the obtained Young modulus was limited by defining it on the basis of the physical values available in the literature [2,3,4,5,6,7,8,9,10,11,12,13].

The proposed optimization procedure contains the following steps:

(1) The sampling of variables;
(2) A parallel numerical analysis of five samples using the Newton–Raphson scheme (analysis);
(3) The acquisition of the force–displacement curves and error norm calculation;
(4) Optimization stage.

**Figure 12:**
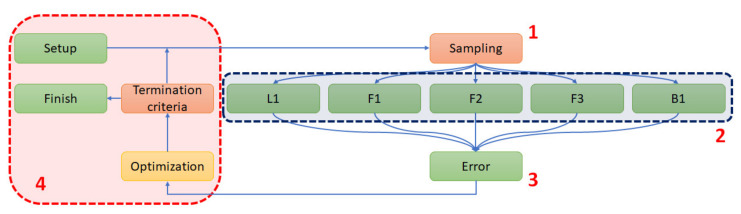
Optimization procedure: (**1**) sampling of variables, (**2**) parallel numerical analysis of five samples, (**3**) calculation of error norms, and (**4**) optimization setup.

The solution was obtained iteratively until the termination criteria were reached (maximum number of repeated solutions) (Figure 12).

To solve this task, direct optimization based on genetic algorithms was used (without the metamodel or response surface approximation). A population size of 200 was arbitrarily chosen, and the maximum number of generations was 30.

Each individual optimization took 3 min and 46 s. A total of 3400 calculations were generated. Simultaneously, 10 calculations were performed in parallel on 24 cores.

## 3. Results

### 3.1. Optimization

In the whole procedure, 17 generations of the population were computed until termination criteria were reached (the number of repeated solutions in subsequent generations). The optimization procedure generated 3400 sample models with varying Young modulus for each density set. From all feasible solutions, an optimal set of parameters was obtained.

**Figure 13:**
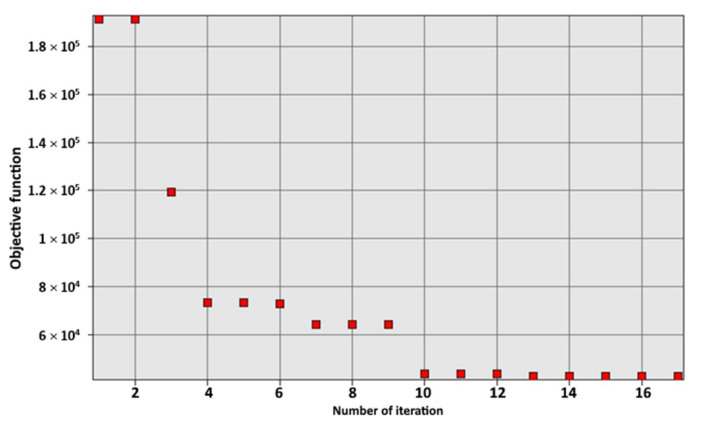
Results of the optimization procedure.

The graph in Figure 13 shows the relationship between the number of iterations and the objective function (with as little error as possible). The graph shows that after 13 iterations the optimization reached a level that could no longer be improved.

**Figure 14:**
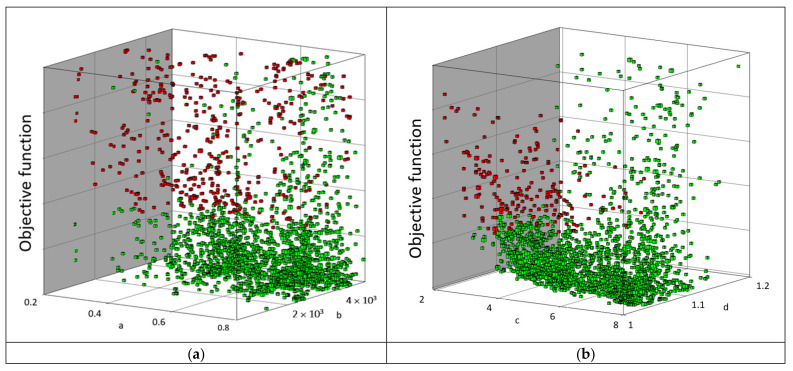
Results of the optimization procedure—coefficients (Equations (1) and (2)): (**a**) (a,b) and (**b**) $\left(c,d\right)$.

The next graph after optimization shows the relationship between coefficients *a*, *b*, *c*, and *d* and the error sum (Figure 14). The red points on the graph are the criteria that do not meet the requirements, while the green points are those that meet the assumed criteria. It can be seen that, for the coefficient *a,* the largest cluster of optimal solutions is in the region of 0.65, while 0.388524 is the most optimal solution. It should be noted that the optimal values of the coefficients a*, b*, c* and d* (Table 7) are, as expected, contained within the domain of the variables and not at their boundaries (Equation (6)).

### 3.2. Method Validation—Step #1

**Figure 15:**
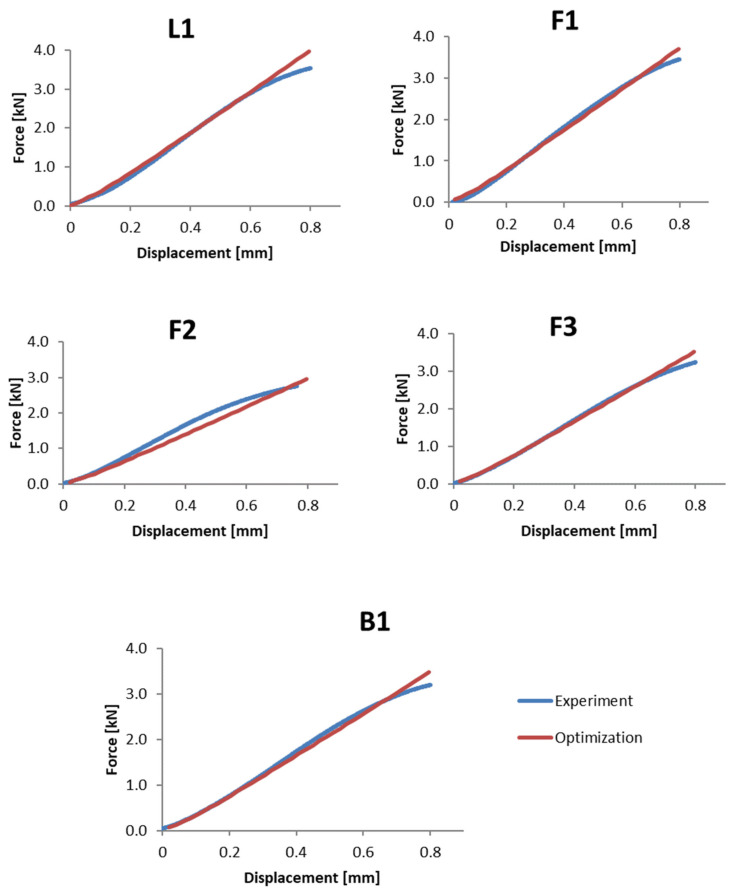
Comparison of the results for the three-point bending test after the numerical optimization of the parameters.

For the set of parameters obtained from the optimization analyses, the consistency of the coefficients for the linear range for individual samples is shown in Table 7. Figure 15 shows the comparative characteristics for the force–displacement relationship of the bone bending tests determined experimentally and numerically. The percentage differences are included in Table 8. The presented results show the acceptable level of convergence between the experimental result and the result obtained from the proposed optimization procedure.

A large convergence of the stiffness attained from the FEM analyses with the stiffness values obtained from the experimental tests can be observed. The differences, especially in the case of F2 and F3, result from a slight deviation in the force displacement curve from the linear characteristic, although the correlation factor $R^{2}=0.994÷0.998$ can be described as very high. This may be due to both local effects at the support and loading points of the samples, and the globally nonlinear behavior of the material. Increasing the accuracy of the results obtained could also be achieved by increasing the number of bone stiffness ranges and reducing the size of a single voxel. However, according to the authors, the discrepancy level below 5% allows the developed methodology for determining bone stiffness based on the grey scale to be considered validated. The individual values of the coefficients are summarized in Table 7.

By substituting the coefficients determined by optimization into Formula (2), the following equation has been obtained:

(7) $$E=0.388524\cdot \;\rho_{app}{}^{1.17823}\;\;where\;\rho_{app}=2.20939\cdot HU+4419.3$$

Based on the data presented above, it is possible to calculate the density of the Young modulus for each range of bone stiffness using Equation (2). The obtained values are presented in Table 9.

### 3.3. Method Validation—Step #2

**Figure 16:**
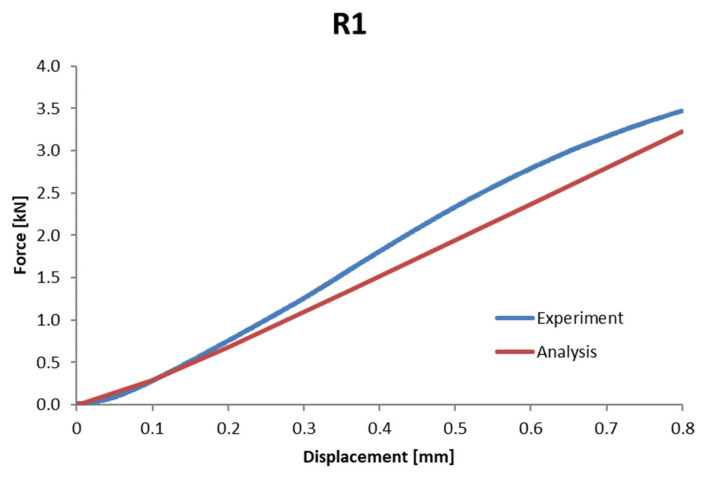
Comparison of the stiffness obtained from the experiment and the numerical analysis for the R1 sample based on the data obtained from the optimization.

The models and mechanical properties of the material, determined on their basis for individual bone components (density ranges), can also be used to describe the behavior of each sample not participating in the optimization procedure presented for a given individual. The comparison of the resulting force–displacement waveforms for sample 6 (R1), which was not involved in optimization, served as an additional verification of the algorithm to determine the stiffness based on the methodology of creating voxel models and optimization (Figure 16). For the R1 sample, an appropriate numerical model was prepared based on the determined parameters *a*, *b*, *c,* and *d*, and it was then used to map the experimental study. In this case, the error in mapping the mean stiffness was 7.0% (Table 10). However, it is also worth noting that, in the case of this sample, the nature of the force–displacement characteristic curve of the bending test differed from the linear course. The correlation factor for this curve was $R^{2}=0.996$.

## 4. Conclusions

The article presents successive stages of determining the stiffness of bone structure for individual ranges of different density. The studies conducted were limited to the linear characteristics and elastic range of the tested materials. They concern only the compact part of the bone.

The voxel models obtained on tomography required rediscretization because the orientation of the voxels was different from the orientation of the basic planes of the cuboidal sample subjected to bending tests. The sample models were modified by determining the midpoints of the individual voxel fractions, creating a new aligned FE mesh representing the real measured dimensions of the samples, and finally mapping the voxels distribution to create individual components with different stiffnesses based on the locations of voxel midpoints. Furthermore, flat surfaces of the walls of the sample models in contact with the supports were obtained, which ensured stable bending analyses, avoiding problems with point contact for the initially determined voxel models.

The use of optimization techniques allowed for the analysis of many stiffness variants of the samples in a short time, and the use of genetic algorithms resulted in the minimization of the stiffness mapping error for all five samples participating in the correlation of the material model. The discrepancies were lower than 5%, which should be considered a satisfactory result with such a heterogenous center and samples taken from different parts of the bone. Finally, the determined data were validated for the next sample that did not participate in the material parameter correlation procedure. After the bending analysis, the stiffness was 7% lower than that obtained experimentally. The authors consider this result to be acceptable considering the complexity of the bone structure.

Generally, it should be considered that the proposed method to determine the parameters of the bone model and, on this basis, for determining its stiffness based on the Hounsfield scale was designed correctly. The values of the Young modulus achieved during the numerical analysis are within the literature range given in Table 1.

In the subsequent research steps, the authors propose to conduct testing of the developed models under the conditions of the stiffness test carried out with the use of the Vickers microhardness tester. Vickers hardness measurements have been found to be very useful for material evaluation, quality control of the manufacturing process, and research and development. Hardness, although empirical in nature, can be correlated with the tensile strength of many metals and is an indicator of wear resistance and ductility. The measurement by the Vickers method is also endowed with the lowest measurement uncertainty. Taking into account the above facts, the authors plan to conduct experimental and numerical tests that will provide the opportunity to validate the presented procedure and the results obtained for the elastic range to develop a constitutive description methodology for the numerical modeling of the behavior of the bone material for the inelastic (nonlinear) range. Certainly, it will also be possible to develop hierarchical bone modeling on this basis, which will require a multiscale approach, for example, to be able to design bone implants on this basis.

## Figures and Tables

**Table 1:** Comparison of mechanical properties of human bones [2,3,4,5,6,7,8,9,10,11,12,13].

Tissue	Young Modulus [GPa]	Poisson Ratio [-]	Density [kg/m^3^]
bone (compact) [3]	20.0	0.37	-
bone (compact) [4]	15.0	0.30	2000.0
bone (compact) [5]	14.0	0.30	-
porous tissue [6]	2.0	-	-
bone (compact) [7]	20.0	0.30	-
bone (compact) [8]	10.5	0.30	-
porous tissue [8]	1.29	0.30	-
bone (compact) [9]	13.7	0.30	-
porous tissue [9]	7.93	0.30	-
tooth [9]	20.0	0.30	-
bone (compact) [10]	16.7	0.30	1750.0
bone (compact) [11]	20.0	0.30	-
bone (compact) [12]	13.8	0.30	-
bone (compact) [13]	13.7	0.38	-
bone (compact) [14]	13.7	0.30	-
porous tissue [14]	0.5	0.30	-

**Table 2:** Dimensions, mass properties, and support spacing for samples in the three-point bending test.

Sample Number	Dimension *A* (Height) (mm)	Dimension *B* (Thickness) (mm)	*L* (Length) (mm)	Cross-Sectional Area *P* (mm^2^)	Moment of Inertia on the Bending Plane *I* (mm^4^)	Bending Strength Index *W* (mm^3^)	Distance between Supports *Δ* (mm)
F1	11.58	7.39	68.14	85.58	956.29	165.16	46
F2	11.35	6.68	70.73	75.82	813.92	143.42	46
F3	11.46	7.20	68.78	82.51	903.04	157.60	46
R1	11.56	7.34	70.10	84.85	944.91	163.48	46
L1	11.58	7.38	68.49	85.46	954.99	164.94	46
B1	11.59	7.39	69.25	85.65	958.77	165.45	46
B2	11.17	7.33	67.80	81.88	851.30	152.43	46
Mean	11.47	7.244	69.04	83.11	911.89	158.93	46
Standard deviation	±0.15	±0.24	±0.98	±3.30	±54.07	±7.71	-

**Table 3:** Parameters of Zwick Roell Kappa.

Manufacturer	Testing Machine	Test Load, Max.	Test Speed Range	Accuracy of the Test Speed	Position Transducer Travel Resolution
Zwick/Roell	Kappa 50 DS	50.0 kN	0.001 mm/h to 100.0 mm/min	<±0.1%	0.068 nm

**Table 5:** Parameters of CT images.

Resolution	Size of a Single Pixel	Distance between Scans	Field of View
793 × 793 pixels	0.15 mm	0.15 mm	118.95 × 118.95 mm

**Table 6:** Percentage distribution of voxels in the sample.

Values According to the Hounsfield Scale *HU*	Percentage of Individual Ranges According to the Hounsfield Scale					
	L1	F1	F2	F3	B1	R1
<600	8.96%	11.22%	13.06%	9.95%	8.16%	14.15%
600–800	3.84%	2.94%	6.17%	1.38%	1.89%	2.07%
800–1000	4.85%	3.61%	7.85%	1.88%	3.18%	2.65%
1000–1200	5.39%	7.65%	12.06%	2.99%	4.35%	3.74%
1200–1400	5.99%	20.78%	16.95%	6.05%	5.02%	6.17%
1400–1600	7.38%	27.32%	23.93%	23.14%	7.14%	18.67%
1600–1800	17.87%	18.66%	15.23%	34.48%	16.52%	34.34%
1800–2000	23.55%	5.95%	2.99%	12.98%	26.79%	18.71%
2000–2200	15.74%	1.33%	1.16%	4.14%	19.35%	4.97%
2200–2400	5.84%	0.37%	0.52%	1.83%	5.51%	0.94%
>2400	0.57%	0.18%	0.08%	1.20%	2.09%	0.36%

**Table 7:** Optimal values of the coefficients that describe stiffness.

Coefficients	Value
a*	0.388524
b*	4.419.3
c*	2.20939
d*	1.17823

**Table 8:** Comparison of sample stiffness with the parentage difference.

Sample	Stiffness Determined from the Experiment $k^{Exp}$ (N/mm^2^)	Stiffness Determined from Optimization $k^{FEA}$ (N/mm^2^)	Difference (%)
L1	5263.7	5259.9	0.072%
F1	4897.4	4905.1	−0.157%
F2	4080.1	3901.9	4.368%
F3	4643.8	4543.9	2.151%
B1	4569.3	4585.8	−0.361%

**Table 9:** Bone density with Young’s modulus for individual density ranges.

Values According to the Hounsfield Scale *HU*	Middle Value	Bone Density *ρ* (kg/m^3^)	Young Modulus *E* (MPa) (Calculated)	Percentage of Particular Layer in the Total Sample (%)	L1	F1	F2	F3	B1
					Number of Voxels				
<600	500	5523.995	9969.031	10.27%	19,923	24,831	25,767	20,777	16,743
600–800	700	5965.873	10,915.16	3.24%	8533	6513	12,174	2880	3870
800–1000	900	6407.751	11,873.88	4.27%	10,781	7992	15,478	3920	6524
1000–1200	1100	6849.629	12,844.46	6.49%	11,991	16,921	23,785	6237	8931
1200–1400	1300	7291.507	13,826.27	10.96%	13,319	45,989	33,445	12,629	10,301
1400–1600	1500	7733.385	14,818.75	17.78%	16,406	60,468	47,211	48,317	14,645
1600–1800	1700	8175.263	15,821.39	20.55%	39,732	41,309	30,040	72,013	33,888
1800–2000	1900	8617.141	16,833.75	14.45%	52,351	13,160	5909	27,112	54,958
2000–2200	2100	9059.019	17,855.4	8.34%	35,001	2933	2294	8643	39,687
2200–2400	2300	9500.897	18,885.98	2.81%	12,989	812	1027	3816	11,302
>2400	2500	9942.775	19,925.13	0.82%	1274	397	166	2504	4279
Mean		7733.385	15,360.02		222,300	221,325	197,296	208,848	205,128

**Table 10:** Comparison of stiffness for sample R1.

Sample	Stiffness Determined from the Experiment $k^{Exp}$ (N/mm^2^)	Stiffness Determined from Optimization $k^{FEA}$ (N/mm^2^)	Difference (%)
R1	4682.2	4353.6	7.02%

## Data Availability

Not applicable.
